# The Developing Brain and Emotion Regulation - Implications for Psychopathology

**DOI:** 10.1192/j.eurpsy.2022.134

**Published:** 2022-09-01

**Authors:** C. Nosarti

**Affiliations:** Department of Child and Adolescent Psychiatry, Institute Of Psychiatry, Psychology And Neuroscience, London, United Kingdom

**Keywords:** preterm birth, brain development, emotion regulation, Psychopathology

## Abstract

In this talk I will describe a series of studies conducted at the Centre for the Developing Brain, King’s College London, that seek to increase our understanding of why infants who are born very early (before 32 weeks’ gestation) are more likely to develop socio-emotional problems when they grow up compared to infants who are born at term. As part of the Evaluation of Preterm Imaging study we carried out multimodal MRI at term in over 200 newborns and studied whether we could identify specific patterns of brain development in those infants who might develop problems with emotion regulation and general mental health as they grow-up. At the behavioural level, we found that very preterm children compared to term-born controls had more mental health problems, including anxiety and autism-spectrum behaviours. Preterm children had lower IQ, were less able to regulate their emotions and inhibit unwanted behaviours. Children’s tendency to attribute negative emotions to daily events, which could lead to increased anxiety, was associated with two main neonatal brain features. These were: 1) weaker structural connectivity in a long-range white matter projection tract called the uncinate fasciculus which connects the frontal lobe with the anterior temporal lobe and 2) altered fronto-limbic functional connectivity, both of which play a critical role in several aspects of social and emotional development. These findings show that early brain changes can be used to predict children’s social and emotional outcomes, hence could be used to inform preventative interventions aimed at averting and targeting emerging emotional disorders.

**Disclosure:**

No significant relationships.

